# PCSK9 Modulates Macrophage Polarization-Mediated Ventricular Remodeling after Myocardial Infarction

**DOI:** 10.1155/2022/7685796

**Published:** 2022-07-04

**Authors:** Feifei Wang, Min Li, Aidong Zhang, Hairui Li, Can Jiang, Jun Guo

**Affiliations:** Department of Cardiology, The First Affiliated Hospital, Jinan University, Guangzhou 510630, China

## Abstract

**Background and Aims:**

An increasing number of high-risk patients with coronary heart disease (similar to acute myocardial infarction (AMI)) are using PCSK9 inhibitors. However, whether PCSK9 affects myocardial repair and the molecular mechanism of PCSK9 modulation of immune inflammation after AMI are not known. The present research investigated the role of PCSK9 in the immunomodulation of macrophages after AMI and provided evidence for the clinical application of PCSK9 inhibitors after AMI to improve cardiac repair.

**Methods and Results:**

Wild-type C57BL6/J (WT) and PCSK9^−/−^ mouse hearts were subjected to left anterior descending (LAD) coronary artery occlusion to establish an AMI model. Correlation analysis showed that higher PCSK9 expression indicated worse cardiac function after AMI, and PCSK9 knockout reduced infarct size, improved cardiac function, and attenuated inflammatory cell infiltration compared to WT mice. Notably, the curative effects of PCSK9 inhibition were abolished after the systemic depletion of macrophages using clodronate liposomes. PCSK9 showed a regulatory effect on macrophage polarization in vivo and in vitro. Our studies also revealed that activation of the TLR4/MyD88/NF-*κ*B axis was a possible mechanism of PCSK9 regulation of macrophage polarization.

**Conclusion:**

Our data suggested that PCSK9 modulated macrophage polarization-mediated ventricular remodeling after myocardial infarction.

## 1. Introduction

Proprotein convertase subtilisin/kexin9 (PCSK9) is the ninth member of the proprotein convertase family, and it has a specialized function of targeting LDL receptor (LDLR) for degradation [1, 2]. The present study showed that the liver synthesized most circulating PCSK9 in peripheral blood [3]. The PCSK9-LDLR complex is transported to the lysosome for degradation and prevents the LDLR from recycling to the cell membrane [4]. Therefore, the functions of PCSK9 in lipoprotein metabolism have attracted increasing attention [5, 6]. Beyond that, PCSK9 has been detected in various tissues in the body, such as the lung [7], brain, and heart [8]. More and more studies concentrated on the role of PCSK9 beyond plasma LDL regulation. In recent years, many studies have found that PCSK9 inhibitors not only can effectively reduce LDL but also are related to early plaque formation, late plaque rupture, thrombosis, and angiogenesis [9]. Sun et al. [10] indicated that PCSK9 interacts with apolipoprotein B and prevents its intracellular degradation irrespective of the low-density lipoprotein receptor. A clinical trial showed that independently of LDL plasma levels, PCSK9 levels correlate with an elevated probability of future cardiovascular events [11]. Many researchers also found that PCSK9 modulated inflammation levels [12, 13] and regulated the levels of inflammatory markers of macrophages, such as Arg-1 and IL10 [13]. Clinical analysis showed that PCSK9 levels correlate with white blood cell count in patients with stable coronary artery disease [14] and PCSK9 affected rheumatoid arthritis and sepsis [15, 16].

Acute myocardial infarction (AMI) is one of the most common reasons for death and heart failure worldwide [17]. In AMI, reduced blood flow to a region of the myocardium led to a region of mechanical weakness [18]. Scar deposition was needed to prevent myocardial rupture and limit functional deterioration in this mechanical weakness myocardium [19]. In the early stage of AMI, this adaptive remodeling is necessary. However, excessive and progressive ventricular remodeling would alter the ventricular structure and cardiac function and eventually lead to the clinical syndrome of HF (heart failure) [20]. The main feature of the intense inflammatory reactions triggered by AMI is the infiltration of leukocytes into the infarcted heart [21]. Numerous studies demonstrated that highly focused inflammatory reactions in infarcted hearts are the main causes of severe complications, including postinfarction heart failure and cardiac rupture [22–24]. Maintaining the balance of adaptive and maladaptive remodeling is very important for the prevention of complications after AMI [25]. Macrophages are pleiotropic cells in the innate immune system and play central roles in the initial inflammatory response to injury and subsequent healing of tissue damaged by ischemia. Macrophages are generally classified into two major subsets: the inflammatory M1 type and the anti-inflammatory M2 type [26]. M1 macrophages are characterized by the secretion of proinflammatory cytokines and growth factors and typically facilitate degradation of the extracellular matrix and remove cell debris during the early phase of myocardial infarction [27, 28]. However, the prolonged presence of M1 macrophages leads to excessive or persistent inflammation and expansion of the infarction area [29]. In contrast, M2 macrophages are characterized by the secretion of anti-inflammatory and reparative factors that facilitate angiogenesis and repair myocardial lesions [30]. Therefore, switching the macrophage phenotype from M1 to M2 is a promising approach to identifying novel therapeutic targets for myocardial repair after myocardial infarction [31–33].

With the wide use of PCSK9 inhibitors in high-risk patients with coronary heart disease (e.g., AMI), researchers are increasingly interested in whether PCSK9 affects myocardial repair after AMI. Emerging evidence indicated that PCSK9 was upregulated in ischemic myocardium and determined the development of infarct size, heart function, and autophagy. However, some studies found that PCSK9 deficiency impacted cardiac lipid metabolism and contributed to the development of HFpEF (HF with preserved ejection fraction) [34]. Therefore, our research further examined the relationship between PCSK9 expression and cardiac function after AMI. The results suggested that PCSK9 gene and protein expression was significantly increased after AMI, and high PCSK9 levels indicated poor cardiac function. The inhibition of highly expressed PCSK9 improved cardiac function. To examine the correlating mechanism, we injected clodronate liposomes to clear systemic macrophages in mice. Notably, the curative effects of PCSK9 inhibition were abolished after macrophage depletion. Based on these research results, we hypothesized that high PCSK9 expression after AMI would lead to poor myocardial repair by promoting M1 macrophage polarization. Inhibition of PCSK9 expression may induce switching of the macrophage phenotype from M1 to M2 and promote myocardial repair after infarction.

## 2. Materials and Methods

### 2.1. Animals

C57BL/6 wild-type mice were purchased from Jiangsu Jicui Yaokang Animal Corp. PCSK9 heterozygous mice on a C57BL/6 background were kindly provided by Dr. Lu Xifeng (Shenzhen University, China) and purchased from the Jackson Laboratory (number 005993-PCSK9^tm1Jdh^). Colonies were maintained by intercrossing heterozygous mutant mice to generate PCSK9^+/+^ and PCSK9^−/−^ mice. All laboratory animal experiments and maintenance procedures were approved by the Institutional Ethics Committee of Guangdong Pharmaceutical University.

### 2.2. Animal Experimental Protocol

Only 10-week-old male mice were used in our study. To create an AMI state, WT and PCSK9^−/−^ mice were anesthetized via 1.5% isoflurane inhalation, and the left anterior descending branch (LAD) was ligated using 7-0 silk suture. The LAD was only threaded in the sham group and was not ligated. The mice were euthanized at 7 days, and the hearts were collected for further examination. To study the role of macrophages, clodronate liposomes were injected (150 *μ*L, 5 mg/mL) (Liposoma BV, NL) into the mouse tail vein 24 h before and after the artery ligation surgery, and mice in the control group were injected with PBS.

### 2.3. Echocardiography

Seven days after surgery, ecthocardiography was performed to assess the left ventricular function of the mice in each group. We used a 12 MHz probe (VisualSonics Vevo 2100, Canada) to perform M-mode and B-mode echocardiography on mice anesthetized via isoflurane (1%) inhalation. The left ventricular ejection fraction (LVEF) and left ventricular fractional shortening (FS%) were quantified as FS% = [(LVEDd − LVESd)/LVEDd] × 100%; LV end-systolic diameter (LVIDs) and LV end-diastolic diameter (LVIDd) were measured.

### 2.4. ELISA of PCSK9

A mouse PCSK9 ELISA kit (BOSTER, China) was used to measure the PCSK9 levels in peripheral blood that was collected 7 days after surgery.

### 2.5. Histological and Immunohistochemical Analysis

Mice were sacrificed with injections of 1% sodium pentobarbital into the cavum abdominis. The chest was opened quickly, perfused, and fixed with 4% paraformaldehyde. 4% paraformaldehyde was used to fix the hearts overnight; then, the hearts were sectioned for H&E staining, immunohistochemical analysis, and Masson trichrome histopathology analyses. The cardiac macrophage population was stained with the macrophage marker F4/80 (Abcam, ab100790, UK). Macrophage polarization marker CD206 and iNOS immunofluorescence antibodies were purchased from Abcam (ab64693, ab3523, UK), and F4/80 antibody was provided by Servicebio (GB11027, China).

### 2.6. Cell Culture and Treatments

Mouse RAW264.7 cells (Procell Life Science & Technology, China) were cultured in 10% FBS high-glucose complete DMEM in 95% air and 5% CO_2_ at 37°C. The RAW264.7 cells were stimulated for 12 h with 1 ng/mL LPS (Sigma, L2880, USA) and for 24 h with 20 ng/mL IL4 (Sigma, SRP3211, USA) to induce M1 macrophage and M2 macrophage differentiation respectively. To investigate the effects of PCSK9 on macrophage polarization, recombinant mouse PCSK9 protein (500 ng/mL) (Novoprotein, CA86, China) was added to polarized RAW264.7 cells for 24 h. To further analyze whether TLR4 is involved in PCSK9-regulated macrophage polarization, cells were pretreated with TLR4 inhibitor (TAK-242, 20 nM) (Sigma, A3850, USA) for 6 h.

### 2.7. Western Blot Analysis

Protein from the mouse hearts and RAW264.7 cells was prepared using a RIPA lysis buffer system (Santa Cruz, CA, USA). A BCA protein assay kit was used to determine the quantity of the protein samples. After the proteins were transferred to a polyvinylidene fluoride membrane and blocking with 5% nonfat milk for 2 h, the membranes were incubated overnight with primary antibody at 4°C, then incubated with a secondary antibody (goat anti-rabbit IgG) for 1 h after washing with TBST. Signals were detected using a Bio-Rad Gel Doc EZ imaging system (Gel Doc EZ Imager, CA, USA). The following primary antibody information was used: PCSK9 (Abcam, ab32727, US), IL6 (CST, 12912, USA), TNF-*α* (CST, 11948, USA), iNOS (CST, 13120, USA), TGF-*β* (CST, 41896, USA), TLR4 (CST, 14358, USA), MyD88 (CST, 4283, USA), and NF-*κ*B (CST, 3036, USA).

### 2.8. Real-Time Quantitative PCR

RNA was extracted from mouse hearts and RAW264.7 cells using the TRIzol reagent and reverse-transcribed using SuperScript II (Life Technologies, USA) at 42°C. The expression of each gene of interest was measured using SYBR Green PCR core reagents (Applied Biosystems). GAPDH was used as an internal mRNA standard, and the 2^–*ΔΔ*^CT method was used to measure the relative expression levels of each gene. Primers were designed according to the following GenBank database information: IL6 (AAGTCCGGAGAGGAGACTTC TGGATGGTCTTGGTCCTTAG), TGF-*β* (CGGAGAGCCCTGGATACCA CGGAGAGCCCTGGATACCA), iNOS (TCACCTTCGAGGGCAGCCGA TCCGTGGCAAAGCGAGCCAG), CD206 (CTGCAGATGGGTGGGTTATT GGCATTGATGCTGCTGTTATG), GAPDH (AGAACATCATCCCTGCCTCTACT GATGTCATCATATTTGGCAGGTT), and PCSK9 (ATGAGCAGTGACCTGTTGGG TGGGCGAAGACAAAGGAGTC).

### 2.9. Flow Cytometry Analysis

Flow cytometry of three fluorescence markers was used to count the percentages of M1 and M2 macrophages in each group after the cells were treated with LPS or IL4 and recombinant mouse PCSK9 protein. M1 macrophages were identified as F4/80^+^/iNOS^+^/CD206^−^ cells, and M2 macrophages were identified as F4/80^+^/iNOS^−^/CD206^+^ cells. After these experimental steps, the RAW264.7 cells were digested into a single-cell suspension at a density of 1 × 10^6^ cells/mL. A 100 *μ*L cell suspension was incubated with fluorescent antibodies (anti-F4/80, anti-iNOS, and anti-CD206) at 4°C for 1 h. The cells were immediately analyzed using flow cytometry. The following primary antibodies were used: CD206 and iNOS immunofluorescence antibodies were purchased from Abcam (ab64693,ab3523, USA), and F4/80 antibodies were provided by Servicebio (GB11027, China).

## 3. Statistical Analysis

Data from at least three independent experiments are presented as the means ± SD, and significant differences between two groups were determined using unpaired *t*-tests. One-way analysis of variance (ANOVA) was used to assess the differences between multiple comparisons followed by Tukey's multiple comparisons test. Two different interventions between multiple comparisons were tested using two-way ANOVA followed by Bonferroni's multiple comparisons test. GraphPad Prism software was used to perform all analyses, and a *P* value less than 0.05 was considered statistically significant.

## 4. Results

### 4.1. Overexpression of PCSK9 in Myocardial Tissue and Peripheral Mouse Blood after AMI

**Figure 1:**
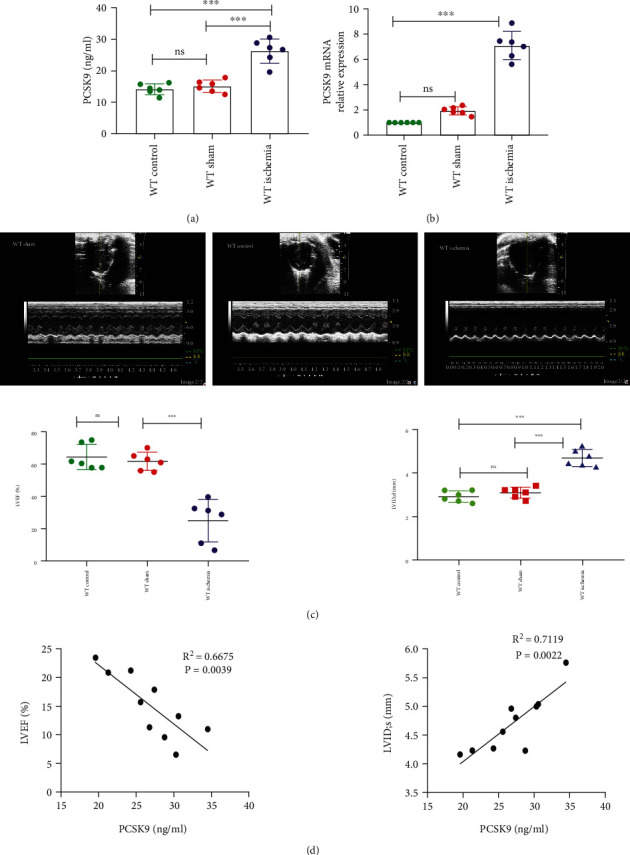
High expression of PCSK9 after acute myocardial infarction and the relationship between cardiac function. (a, b) Compared with WT control and WT sham group, the mice after AMI have a high level of PCSK9 protein and mRNA. (c) LVEF% and LVIDd measured by echocardiography 7 days after AMI, *n* = 6. (d) The correlation between the level of PCSK9 protein and cardiac function EF%, LVIDs in the mice after AMI, *n* = 10. ^∗^*P* < 0.05; ^∗∗^*P* < 0.01; ^∗∗∗^*P* < 0.001; ns: not significant.

To clarify the changes in PCSK9 expression after myocardial infarction, q-PCR and ELISA were performed. The ELISA analysis showed that the expression of PCSK9 in peripheral mouse blood was increased in the WT ischemia group compared to the control group and WT sham group (*P* < 0.05) (Figure 1(a)). The results also showed that PCSK9 mRNA expression in the WT ischemia group was obviously increased compared to the control group and WT sham group (*P* < 0.05) (Figure 1(b)). To further understand the relationship between PCSK9 expression and cardiac function after AMI, EF% and LVIDd were measured using echocardiography 7 d after AMI (Figure 1(c)), and the correlation between these factors was calculated. The results showed that PCSK9 expression is negatively correlated with the LVEF (*R*^2^ = 0.6675, *P* = 0.0039) and positively correlated with the LVIDs (*R*^2^ = 0.7119, *P* = 0.0022) (Figure 1(d)). Taken together, the data showed that the expression of PCSK9 increased significantly after AMI in mice. The correlation analysis indicated a significant correlation between PCSK9 expression and cardiac function after myocardial infarction.

### 4.2. Inhibition of Highly Expressed PCSK9 Reduced Infarct Size and Inflammation and Improved Heart Function after AMI in Mice

**Figure 2:**
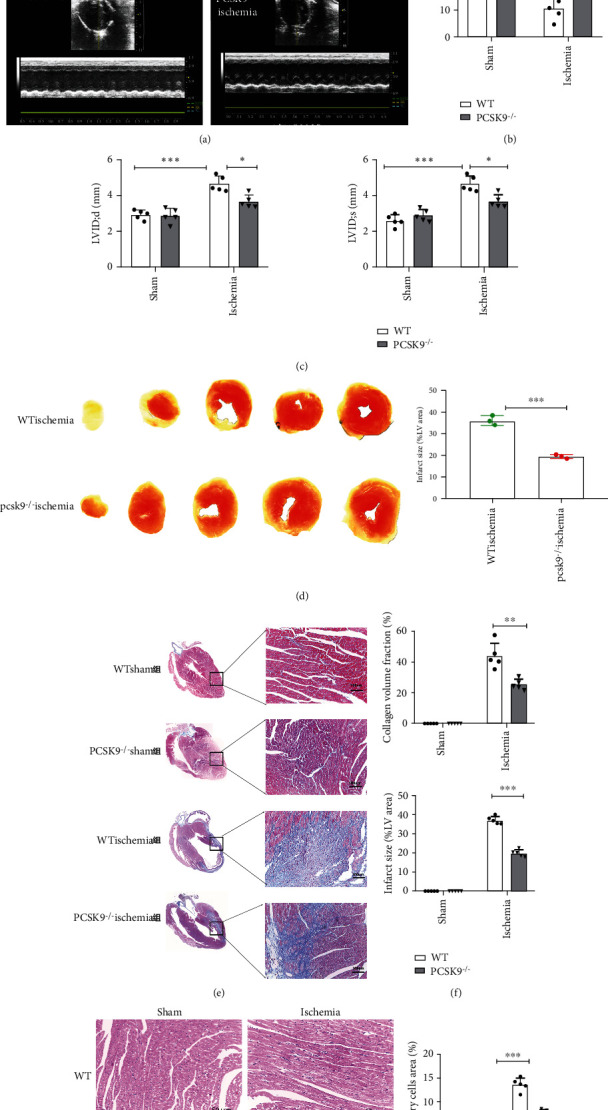
Inhibition of highly expressed PCSK9 reduced infarct size, inflammation, and myocardial fibrosis and improved cardiac function after 7 days of AMI. (a–c) Cardiac function measured by echocardiography, *n* = 5. (d) TTC staining showed the infarct size and quantitative analysis by ImageJ in each group, *n* = 5. (e) Masson staining for infarct size and myocardial fibrosis. Scale bar = 1 *μ*m, *n* = 5. (f) Quantitative analysis by ImageJ for collagen volume fraction and percentage infarct size of hearts in (e). (g) HE staining for the infract regions in hearts. Scale bar = 50 *μ*m, *n* = 5, and quantification of inflammatory cell infiltration. ^∗^*P* < 0.05; ^∗∗^*P* < 0.01; ^∗∗∗^*P* < 0.001; ns: not significant.

To inquire about the role of PCSK9 in myocardial injury, we constructed PCSK9-knockout mice. Seven days after LAD artery ligation surgery, we compared the infarct size, inflammation, echocardiographs, and myocardial fibrosis of the mice in the WT and PCSK9^−/−^ ischemia groups. The results of echocardiography showed that LVEF (16.02 ± 6.8% vs. 35.14 ± 5.0%, *P* < 0.05) (Figures 2(a) and 2(b)) and LVFS (10.62 ± 4.09% vs. 18.01 ± 1.16%, *P* < 0.05) (Figures 2(a) and 2(b)) in the PCSK9^−/−^ ischemia group were significantly higher than that in the WT ischemia group. The LVIDd (4.65 ± 0.43 vs. 3.76 ± 0.36, *P* < 0.05) (Figures 2(a) and 2(c)) and LVIDs (4.43 ± 0.33 vs. 3.47 ± 0.18, *P* < 0.05) (Figures 2(a) and 2(c)) were lower than that in the WT ischemia group (*P* < 0.05). The results of TTC staining showed that the infarct size was obviously smaller in the PCSK9^−/−^ ischemia group (36.15 ± 2.24% vs. 19.47 ± 0.91%, *P* < 0.05) (Figure 2(d)). Masson staining was used to evaluate the extent of myocardial fibrosis and indicated that the collagen density in the infarcted area of the PCSK9^−/−^ ischemia group was obviously decreased compared to the WT ischemia group (44.15 ± 8.38% vs. 25.32 ± 3.23%, *P* < 0.05) (Figures 2(e) and 2(f)). HE staining of myocardial tissue showed that a large number of inflammatory cells infiltrated the WT ischemia group myocardium compared to the WT sham group, but the number of inflammatory cells decreased notably when PCSK9 expression was knocked down (13.6 ± 1.39% vs. 7.50 ± 0.81%, *P* < 0.05) (Figure 2(g)). These figures suggested that inhibition of highly expressed PCSK9 after AMI reduced the infarct size, myocardial fibrosis, and inflammatory response and promoted cardiac function repair after acute myocardial infarction.

### 4.3. Systemic Depletion of Macrophages Reduced the Benefits of PCSK9 Knockout in Cardiac Repair after Myocardial Infarction

**Figure 3:**
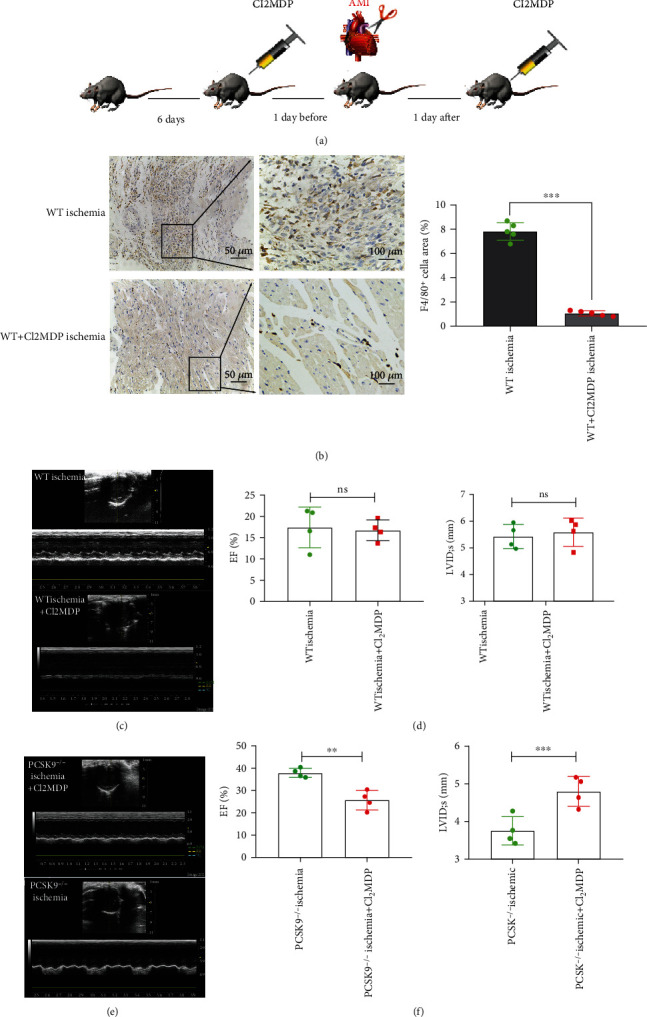
Systemic depletion of macrophages reduced the benefits of PCSK9 knockout in cardiac repair after myocardial infarction. (a) After being adaptively fed for 6 days, Cl_2_MDP or PBS were injected into the tail vein to systemically deplete macrophages. (b) Immunohistochemical staining for F4/80 expression in mouse hearts from Cl_2_MDP- and PBS-treated mice after myocardial infarction. Scale bar = 50 *μ*m, *n* = 5. Quantitative analysis by ImageJ for F4/80^+^ cells of myocardium in (b). (c, d) Cardiac function measured by echocardiography after Cl_2_MDP and PBS treatment in the WT ischemia group, *n* = 4. (e, f) Cardiac function measured by echocardiography after Cl_2_MDP and PBS treatment in the PCSK9^−/−^ ischemia group, *n* = 4. ^∗^*P* < 0.05; ^∗∗^*P* < 0.01; ^∗∗∗^*P* < 0.001; ns: not significant.

The role of macrophages in the modulation of cardiac inflammation after AMI was explored in recent years, but the relationship between macrophages and the effects of PCSK9 on the modulation of cardiac function after AMI are not clear. To examine this relationship, we injected clodronate liposomes into the mouse tail vein 24 h before and after the artery ligation surgery (Figure 3(a)). The results showed that clodronate liposomes significantly reduced the cardiac macrophage population, as indicated by the F4/80 macrophage marker level in immunohistochemistry analyses (Figure 3(b)). After clodronate liposome injection, we found that cardiac function was not significantly different between the Cl_2_MDP-treated and PBS-treated WT ischemia groups in LVEF (17.41 ± 4.76% vs. 16.72 ± 2.45%, *P* > 0.05) or LVIDs (5.43 ± 0.45% vs. 5.59 ± 0.53%, *P* > 0.05) (Figures 3(c) and 3(d)). Clodronate liposomes did not aggravate ischemic injury. However, clodronate liposomes attenuated the benefits of PCSK9 gene knockout, as indicated by the worsened cardiac function. Cardiac function was significantly worse in the Cl_2_MDP-treated group than in the PBS-treated PCSK9^−/−^ ischemia group, including LVEF (37.95 ± 2.01% vs. 15.40 ± 3.22%, *P* < 0.05) and LVIDs (3.75 ± 0.37 vs. 4.80 ± 0.39, *P* < 0.05) (Figures 3(e) and 3(f)). These results showed that depletion of macrophages attenuated the benefits of PCSK9 gene knockout after myocardial infarction. Therefore, cardiac macrophages were required for the effects of PCSK9 on infarction repair.

### 4.4. PCSK9 Knockout Inhibited M1 Polarization and Promoted M2 Polarization in Myocardial Macrophages after Infarction

**Figure 4:**
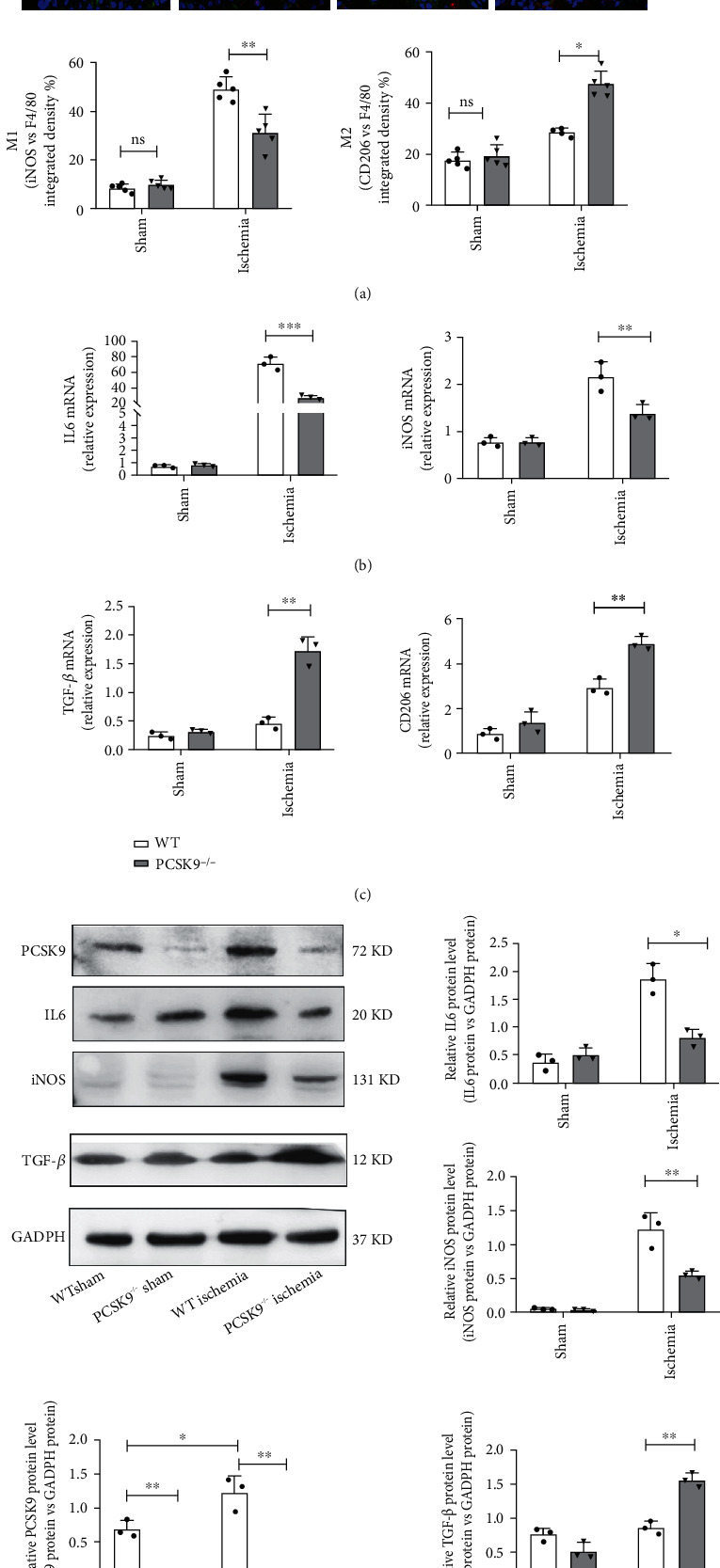
PCSK9 knockout inhibited M1 polarization and promoted M2 polarization in myocardial macrophages after infarction. (a) Representative immunofluorescence staining showing the percentages of M1 (F4/80^+^iNOS^+^CD206^−^) and M2 (F4/80^+^iNOS^−^CD206^+^) in WT/PCSK9^−/−^ mouse myocardium after ischemia or sham. Nuclei were counterstained with DAPI. Scale bar = 50 *μ*m, *n* = 5. Quantitative analysis of the percentage of M1 and M2 macrophages of (a). (b, c) q-PCR analysis of IL-6, iNOS, TGF-*β*, and CD206 mRNA expression in WT/PCSK9^−/−^ mouse myocardium after ischemia or sham, *n* = 3. (d) Representative images of Western blots for PCSK9, IL6, iNOS, and TGF-*β* in WT/PCSK9^−/−^ mouse myocardium after ischemia or sham, *n* = 3.

Macrophages exhibit distinct subtypes and polarization statuses after infarction. On the basis of our results, we investigated the function of PCSK9 on cardiac macrophage polarization in mice after infarction. The proportions of M1 (F4/80^+^iNOS^+^CD206^−^) and M2 (F4/80^+^iNOS^−^CD206^+^) macrophages were measured using immunofluorescence staining of the myocardium. The results showed that the proportion of M2 macrophages was increased compared to the WT ischemia group (27.58 ± 0.97% vs. 43.34 ± 0.61%, *P* < 0.05) (Figure 4(a)), and the proportion of M1 macrophages was decreased in the PCSK9^−/−^ ischemia group (45.32 ± 4.19% vs. 34.54 ± 2.95%, *P* < 0.05) (Figure 4(a)). The results of the q-PCR and Western blot analyses showed that the expression of M1 macrophage markers (IL6, iNOS) was remarkably reduced in the PCSK9^−/−^ ischemia group compared to the WT ischemia group (Figures 4(b)–4(d)), but the expression of M2 macrophage markers (TNF-*β* and CD206) was increased (Figures 4(b)–4(d)). Collectively, the results showed that PCSK9 knockout polarized macrophages from the M1-like phenotype toward the M2-like phenotype upon myocardial injury.

### 4.5. In Vitro, Exogenous PCSK9 Protein Induced Inflammatory Macrophages to Acquire the M1 Phenotype

**Figure 5:**
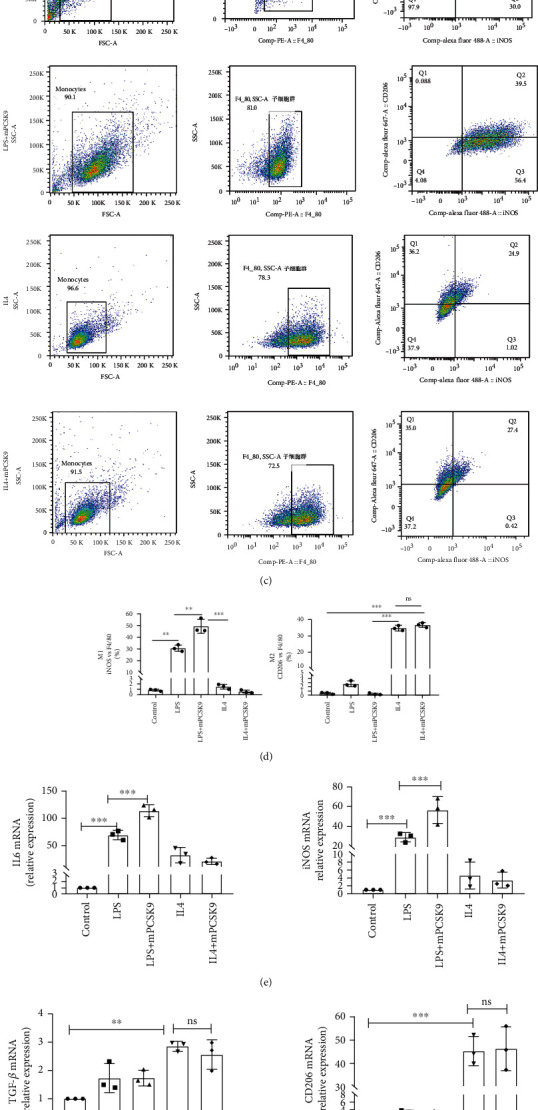
In vitro, the exogenous PCSK9 protein induced inflammatory macrophages to acquire the M1 phenotype. The morphologic changes in macrophages stimulated by LPS/IL4. Cell shape changed from round to fusiform in LPS-stimulated RAW264.7 cells to ellipse in IL4-stimulated RAW264.7 cells. Scale bar = 50 *μ*m. (b) 0.5 *μ*g/mL PCSK9 protein significantly induced IL6 expression in RAW264.7 and have no effect on cell viability. (c) Representative flow cytometry plots showing the percentages of M1 (F4/80^+^/iNOS^+^/CD206^−^) and M2 (F4/80^+^/iNOS^−^/CD206^+^) phenotype in LPS/IL4-stimulated RAW264.7 cells after cocultivation with PCSK9 protein for 24 h, *n* = 3. Pooled flow cytometry data from (c). (e, f) q-PCR analysis of IL-6, iNOS, TGF-*β*, and CD206 mRNA expression in LPS/IL4-stimulated RAW264.7 cells after cocultivation with PCSK9 protein for 24 h, *n* = 3. (g) Representative images of Western blots for IL6, iNOS, and TGF-*β* in LPS/IL4-stimulated RAW264.7 cells after cocultivation with PCSK9 protein for 24 h, *n* = 3. Protein levels of IL6, iNOS, and TGF-*β* of (g). ^∗^*P* < 0.05; ^∗∗^*P* < 0.01; ^∗∗∗^*P* < 0.001; ns: not significant.

To examine the effect of PCSK9 on macrophage polarization in vitro, recombinant mouse PCSK9 protein was added to polarized RAW264.7 cells stimulated with 1 ng/mL LPS and 20 ng/mL IL4 for 24 h (Figure 5(a)). The concentration grading experiment revealed that 0.5 *μ*g/mL PCSK9 protein significantly induced IL6 expression in RAW264.7 cells and had no effect on cell viability (Figure 5(b)). The expression of M1 markers (IL6 and iNOS) and M2 markers (TGF-*β* and CD206) in cultured RAW264.7 cells was determined using q-PCR, Western blotting, and flow cytometry. Flow cytometry analysis showed that the proportion of M1 macrophages increased significantly (*P* < 0.05) (Figures 5(c) and 5(d)), but there was no significant difference in the proportion of M2 macrophages (*P* > 0.05) (Figures 5(c) and 5(d)) compared to the control group. The q-PCR results showed that PCSK9 mRNA addition increased the expression of IL6 and iNOS (*P* < 0.05) (Figure 5(e)), but there were no significant differences in the expression of TGF-*β* and CD206 (*P* > 0.05) (Figure 5(f)) compared to the control group. Western blot analysis also showed that M1 markers (IL6 and iNOS) increased significantly (*P* < 0.05) (Figure 5(g)), but M2 markers (TGF-*β* and CD206) were not significantly different (*P* > 0.05) (Figure 5(g)). These data showed that high expression of PCSK9 promoted M1 macrophage polarization in vitro.

### 4.6. PCSK9 Regulated M1 Macrophage Polarization by Targeting TLR4

**Figure 6:**
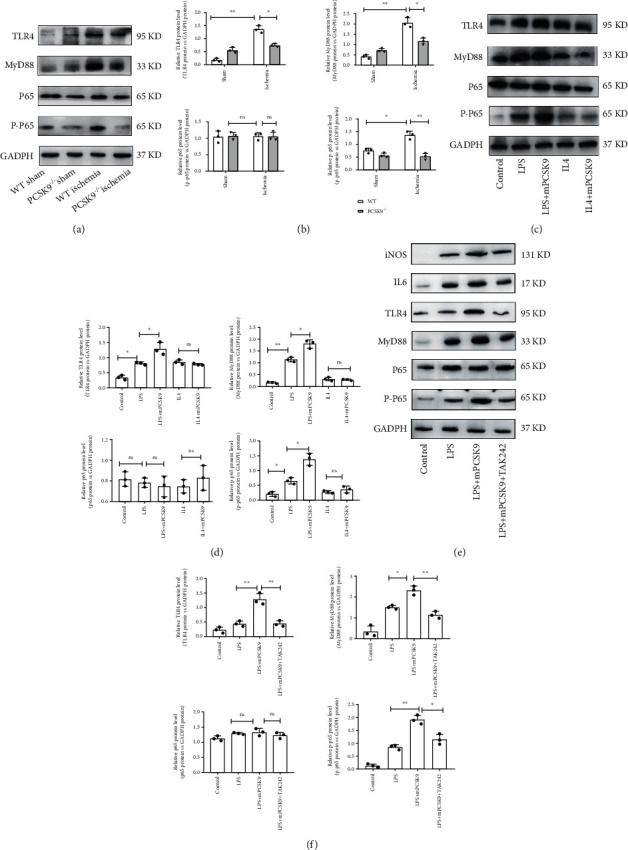
PCSK9 regulated M1 macrophage polarization by targeting TLR4. Representative images of Western blots for TLR4 and downstream MyD88/NF-*κ*B in WT/PCSK9^−/−^ mouse myocardium after ischemia or sham, *n* = 3. (b) Protein levels of TLR4 and downstream MyD88/NF-*κ*B of (a). (c) Representative images of Western blots for TLR4 and downstream MyD88/NF-*κ*B in LPS/IL4-stimulated RAW264.7 cells after cocultivation with PCSK9 protein for 24 h, *n* = 3. (d) Protein levels of TLR4 and downstream MyD88/NF-*κ*B of (c). (e)TLR4 inhibitor (TAK242) was used to analyze whether TLR4 is involved in PCSK9-regulated macrophage polarization; (f) protein levels of IL6, iNOS, TLR4, and downstream MyD88/NF-*κ*B of (e). ^∗^*P* < 0.05; ^∗∗^*P* < 0.01; ^∗∗∗^*P* < 0.001; ns: not significant.

In vivo and in vitro Western blot analyses showed that high expression ofPCSK9 significantly upregulated the TLR4/MyD88/NF-*κ*B pathway. In vivo, the expression of the TLR4/MyD88/NF-*κ*B pathway was upregulated in the WT ischemia group and downregulated in the PCSK9^−/−^ ischemia group (Figures 6(a) and 6(b)). In vitro, after cocultivation with PCSK9 protein for 24 h, TLR4 and downstream MyD88/NF-*κ*B were significantly upregulated in the LPS+mPCSK9 group compared with the LPS group. But there was no significant difference between the IL4 group and the IL4+mPCSK9 group (Figures 6(c) and 6(d)). To further analyze whether TLR4 is involved in PCSK9-regulated macrophage polarization, cells were pretreated with TLR4 inhibitor (TAK-242), TAK-242 is a small-molecule cyclohexene derivative, and it selectively binds to Cys-747 in the TIR (Toll/IL-1 receptor) domain of the intracellular receptor, which in turn hinders the downstream adaptor proteins (TRAM and TIRAP) from binding to TLR4 to totally suppress the pathway [35, 36]. Western blot was performed to detect levels of M1 macrophage markers IL-6 and iNOS. As shown in Figures 6(e) and 6(f), LPS was added to culture systems in order to induce an inflammatory microenvironment. With the presence of inhibitors TAK-242, the M1 macrophage markers iNOS and IL-6 induced by PCSK9 were suppressed and significantly lower than those without inhibitors (*P* < 0.05). Meanwhile, the presence of inhibitors TAK-242 significantly upregulates the level of the MyD88/NF*κ*B pathway. The results suggested that TLR4 may be a key factor in PCSK9-regulated macrophage polarization.

## 5. Discussion

Animal experiments previously showed that the plasma PCSK9 concentration was significantly increased in AMI [37]. SREBP-2 and HNF1*α* are predominant transcription factors for PCSK9 and play important roles in the upregulation of PCSK9. PCSK9 gene and protein expression were significantly increased in the ischemia group 7 d after LAD ligation in our study, which was consistent with previous studies. The correlation analysis between these factors indicated that the PCSK9 level positively correlated with LVIDs and negatively correlated with LVEF. These data suggested that PCSK9 gene and protein expression were significantly increased after AMI, and a high PCSK9 level indicated poor cardiac function. Because of relatively small samples in linearity analysis, larger samples should be used in future studies, particularly clinical samples.

Another interesting finding of our research was that TGF-*β*, which is an important regulator of cardiac fibrosis, was upregulated in the PCSK9^−/−^ group compared to the WT group after AMI, which was not completely consistent with the Masson staining results in our study. We hypothesized that the reason for this difference was that the role of TGF-*β* signaling in the infarcted myocardium always elicited complex and opposing cellular responses [38]. Some studies showed that early TGF-*β* antagonism within 24 h following myocardial infarction led to increased mortality and enhanced proinflammatory cytokine and chemokine gene expression [39]. In contrast, late TGF-*β* inhibition decreased collagen deposition after the infarct healing and meanwhile attenuated adverse remodeling [40]. Therefore, TGF-*β* likely exerts different roles during different stages of myocardial infarction. Our research found that PCSK9 knockout provided better protection against myocardial injury by upregulating TGF-*β* and inducing M2 macrophage polarization. We will design experiments to further demonstrate the effects of PCSK9 in TGF-*β* modulation of fibroblast phenotype and fibrosis. What is the role of high expression of PCSK9 regarding the function and adaptation of cardiomyocytes? The Ding et al. study showed that hypoxia-induced PCSK9 expression in cardiomyocytes and expression of PCSK9 were dependent on the duration of hypoxia; what is more, PCSK9 secretion by cardiomyocytes causes the development of autophagy [8]. According to the abovementioned results, we hypothesized that ischemia-hypoxia-induced cardiomyocytes in ischemic areas secreted PCSK9 which regulated macrophage switching to inflammatory M1 polarization. The macrophage/cardiomyocyte coculture system under hypoxia conditions will be designed to test this hypothesis in future studies.

The relationship between cardiac function after AMI and the levels of PCSK9 in peripheral blood has not been established in clinical studies. Wiviott et al. showed that PCSK9 inhibitors had different effects on different myocardial infarct subtypes [41]. For example, evolocumab (PCSK9 monoclonal antibody) reduced the risk of spontaneous and procedural AMI but had no effect on type 2 (as indicated by a mismatch in myocardial oxygen supply and demand) AMI events [42]. The ORION-4 trial will assess the effects of reduced PCSK9 levels in circulation on clinical outcomes [43]. Recent studies showed that better outcomes were attributed to LDL-C level reduction [44]. Our study is the first report to explain the possible mechanisms by which PCSK9 inhibition modulates the immunoregulatory functions of macrophages to promote heart repair after infarction. Through this study, we wanted to explore the causative role of PCSK9 in post-AMI ventricular remodeling and its potential as a therapeutic target for cardiac repair after AMI.

Macrophages play important roles in the extent and effect of inflammatory cell infiltration in the ischemic heart following AMI [45]. Therefore, promotion of proinflammatory M1 macrophage switching to anti-inflammatory M2 macrophages after AMI may be a novel treatment for immune regulation [18]. The exact mechanisms of macrophage polarization post-MI are not clear [30], and the sources of macrophages and the local microenvironment may explain the difficulty in determining the mechanism of macrophage phenotype switching [27, 46]. The microenvironment post-MI is filled with early proinflammatory M1 factors and anti-inflammatory M2 macrophages, which likely induce macrophage polarization [47, 48]. Previous studies showed that PCSK9 was a direct inflammatory mediator because PCSK9 increased the expression of proinflammatory cytokines, such as TNF-*α* and IL-6, in macrophages [49, 50]. Investigation into the regulatory function of PCSK9 in atherosclerosis showed that PCSK9 activated NF-*κ*B signaling to promote inflammation [12]. The inhibition of TLR4 and its downstream MyD88/NF-*κ*B signaling pathway has been shown to alleviate inflammation by negatively polarizing M1 macrophages [51]. Our research revealed that overexpression of PCSK9 in myocardium and macrophage both upregulated TLR4 and its downstream MyD88/NF-*κ*B expression which also induced M1 macrophage polarization. With the presence of TLR4 inhibitors TAK-242, the M1 macrophage markers induced by PCSK9 were suppressed and significantly lower than those without inhibitors. These results demonstrated that PCSK9 induced M1 macrophage polarization by promoting the activation of the TLR4/MyD88/NF-*κ*B pathway.

In summary, our findings suggest that high expression of PCSK9 after AMI leads to poor myocardial repair by regulating M1 macrophage polarization via TLR4/MyD88/NF-*κ*B signaling. In contrast, PCSK9 knockout provides better protection against myocardial injury by inducing M2 macrophage polarization. However, deficiency of clinical data of patients who used PCSK9 inhibitor after AMI is the limitation of our research. The major reason is that there are only two monoclonal antibodies targeting PCSK9 available for treating hypercholesterolemia in clinic. They can obviously decrease LDL particles in blood without affecting plasma PCSK9 levels. In further studies, we will screen desirable PCSK9 small molecule inhibitors which can reduce plasma PCSK9 levels in vivo to reveal more PCSK9 functions in cardiovascular diseases beyond LDL-cholesterol plasma level regulation.

## Data Availability

The data used to support the findings of this study are included within the article.

## Abbreviations

PCSK9: Proprotein convertase subtilisin kexin 9
LVIDd: LV end-diastolic diameter
EF%: Ejection fraction
FS%: Fractional shortening
LVIDs: LV end-systolic diameter
LPS: Lipopolysaccharide
TLR4: Toll-like receptor 4
Cl_2_MDP: Clodronate liposomes.

## Supplementary Materials

Supplementary Materials Graphical Abstract: high PCSK9 expression after AMI would lead to poor myocardial repair by promoting M1 macrophage polarization; inhibition of PCSK9 expression may induce switching of the macrophage phenotype from M1 to M2 and promote myocardial repair after infarction.
