# Supplementary material for: PCSK9 Modulates Macrophage Polarization-Mediated Ventricular Remodeling after Myocardial Infarction
Source: J Immunol Res. 2022 Jul 4;2022:7685796. doi: 10.1155/2022/7685796 (PMC9273409; doi:10.1155/2022/7685796)
Supplement: Supplementary Materials — Graphical Abstract: high PCSK9 expression after AMI would lead to poor myocardial repair by promoting M1 macrophage polarization; inhibition of PCSK9 expression may induce switching of the macrophage phenotype from M1 to M2 and promote myocardial repair after infarction. [file 7685796.f1.doc]

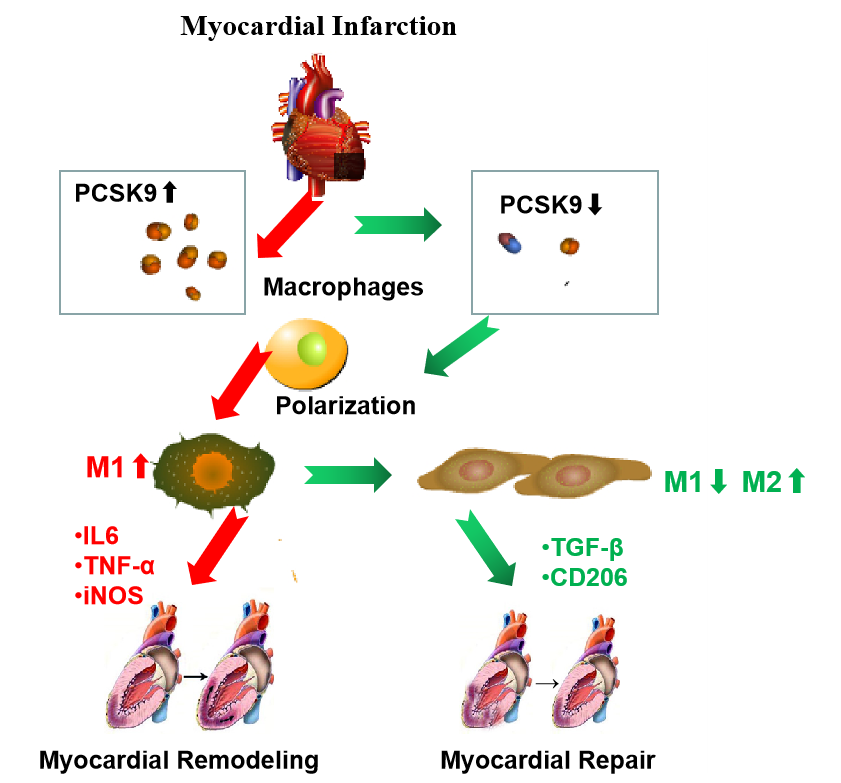


High PCSK9 expression after AMI would lead to poormyocardial repair by promoting M1 macrophage polarization, inhibition of PCSK9 expression may induce switching of the macrophage phenotype from M1 toM2 and promote myocardial repair after infarction.
